# Dementia care providers’ delivery of family caregiver support during COVID-19

**DOI:** 10.1093/geroni/igaa057.3447

**Published:** 2020-12-16

**Authors:** Emma Quach, Emily Franzosa, Rachael Scali, Lauren Moo, Christine Hartmann

**Affiliations:** 1 Holden, Massachusetts, United States; 2 James J. Peters VA Medical Center, Bronx, New York, United States; 3 Bedford VA Research Corporation, Inc., Belmont, Massachusetts, United States; 4 Bedford VA Medical Center, Bedford, Massachusetts, United States; 5 Edith Nourse Rogers Memorial Veterans Hospital, Bedford, Massachusetts, United States

## Abstract

Family caregiver support is a cornerstone of dementia care. Yet the transition to virtual care during COVID raised questions about the ability of dementia care teams to maintain caregiver support services. We surveyed Veterans Affairs clinicians about dementia caregiver support delivery following the COVID surge in the six New England states. 38 out of 68 (55%) clinicians from 6 states responded in June and July 2020. We found: 1) Clinicians continued providing the same types of support services for family caregivers before and after COVID, with over 50% of providers interacting with caregivers daily or multiple times per week. The most prevalent services were caregiver needs assessments, information and referrals, and assistance with accessing services. Two-thirds reported continuing to offer caregiver skills training and counseling, including peer support groups. 2) Caregiver support modality changed, most frequently through the combined use of phone and video, followed by only phone, and rarely, by only video. 3) Providers indicated that phone, more than video, increased to replace in-person interactions, because of multiple factors: caregivers (who continued to call for support on an as-needed basis but declined video encounters), providers (who began to provide group support via phone), and service factors (ad hoc versus scheduled encounters). Results suggest clinicians continued providing caregiver support despite suspension of in-person interactions, but future research is needed to assess the impacts of caregiver support delivery mostly by phone and factors underlying the limited use of video in delivering caregiver support.

